# OA20 More Than Active Disease: Transition in a Vulnerable Young Person with JIA

**DOI:** 10.1093/rap/rkag113.020

**Published:** 2026-11-03

**Authors:** Zahra M Iftikhar, Hassan Butt, Geetha Janakiraman, Lucy Craig, Ethan Sen, Flora McErlane

**Affiliations:** South Tyneside and Sunderland NHS Foundation Trust, Sunderland, United Kingdom; Newcastle Upon Tyne Hospitals NHS Foundation Trust, Newcastle, United Kingdom; South Tees Hospitals NHS Foundation Trust, Middlesbrough, United Kingdom; Newcastle Upon Tyne Hospitals NHS Foundation Trust, Newcastle, United Kingdom; Newcastle Upon Tyne Hospitals NHS Foundation Trust, Newcastle, United Kingdom; Newcastle Upon Tyne Hospitals NHS Foundation Trust, Newcastle, United Kingdom

## Abstract

**Introduction:**

Transition from paediatric to adult rheumatology is recognised as a vulnerable period for young people with juvenile idiopathic arthritis (JIA), particularly where psychosocial complexity coexists with chronic disease. Persistent non-inflammatory pain can complicate management and increase the risk of overtreatment and patient disengagement. It is important to distinguish this from active inflammatory disease. We present a case of longstanding polyarticular JIA in a socially vulnerable adolescent managed within the Northeast regional transition pathway, where preparation for transfer has relied not only on disease control but also structured multidisciplinary support, adolescent-centered care and continuity across paediatric and adult rheumatology services.

**Case description:**

We present a young adult aged 17 with RF-negative, ANA-positive polyarticular JIA. She was diagnosed at age 3 under paediatric rheumatology at the Great North Children’s Hospital (GNCH) following an 8-month history of knee pain and episodic swelling, with involvement of 20 joints. Initial management included intravenous and oral corticosteroids, naproxen, and subcutaneous methotrexate. Ophthalmology review identified one episode of uveitis.

Methotrexate was discontinued after achieving remission in 2015. Subsequent symptom relapse prompted an ultrasound evaluation which confirmed the absence of active synovitis, and symptoms were managed conservatively with physiotherapy and hydrotherapy. Following confirmed relapse later that year, methotrexate was recommenced. Due to adalimumab inefficacy, treatment was escalated to intravenous tocilizumab in 2018.

Repeated clinical assessments identified chronic biomechanical pain alongside intermittent inflammatory disease activity, creating ongoing diagnostic and therapeutic challenges. Tocilizumab and methotrexate were stopped in 2020 but ultrasound-confirmed relapse in 2021 prompted intravenous methylprednisolone and reintroduction of tocilizumab. Subcutaneous methotrexate was restarted in 2022 and treatment later rationalised to subcutaneous tocilizumab every two weeks once disease control was achieved.

Her social background significantly influenced care delivery. Early childhood was affected by maternal alcohol dependency, bereavement, and changes in guardianship. Additional concerns identified through routine assessment included previous self-harm, vaping and sexual health needs. Given methotrexate exposure and transition to adult services, reproductive health counselling formed a routine component of care. She received education regarding dual contraception, pregnancy planning and subsequently engaged with long-acting reversible contraception.

Transition planning commenced from age 12 through adolescent clinics, Ready Steady Go documentation, regular HEADDSS assessment, and MDT involvement. Continuous support from the paediatric rheumatology nurse consultant provided consistency throughout adolescence and will facilitate bridged continuity. Identification as a higher-risk transition patient enabled enhanced oversight, multidisciplinary handover and planned transfer from paediatric rheumatology at GNCH to adult rheumatology at James Cook University Hospital.

**Discussion:**

This case illustrates that transition complexity in JIA extends beyond disease phenotype and treatment burden. Although inflammatory disease required multiple treatment escalations over time, clinical decision-making was repeatedly complicated by coexisting chronic biomechanical pain. Symptoms did not consistently correlate with objective evidence of synovitis, highlighting the importance of integrating imaging, functional assessment, and longitudinal review to avoid both undertreatment and unnecessary immunosuppression.

Equally important was recognition that social vulnerability represented a major determinant of transition risk. Family instability, bereavement, previous mental health concerns and changing living arrangements increased the likelihood of disengagement at a developmentally sensitive stage. Identification of these factors through structured adolescent assessment enabled proactive intervention rather than reactive management following non-attendance or deterioration.

This case also demonstrates the importance of embedding adolescent health promotion within rheumatology practice. Discussions regarding sexual health and contraception may be overlooked during disease-focused consultations but are particularly relevant in young people receiving disease-modifying therapy and approaching transfer to adult care. Developmentally appropriate counselling supported her informed decision-making, medication safety awareness, and increasing healthcare autonomy.

The Northeast transition model supports preparation over several years rather than treating transition as a single transfer event. Age-appropriate adolescent clinics, Ready Steady Go assessment, specialist nurse continuity, multidisciplinary input and access to youth support worker and transition nursing services for vulnerable young people aim to preserve continuity across paediatric and adult services. As this patient approaches transfer, this case highlights the value of coordinated regional transition pathways in promoting engagement and supporting continuity of care for vulnerable young people with chronic rheumatic disease.

**Key learning points:**

1. Persistent musculoskeletal symptoms in JIA should not automatically be assumed to represent inflammatory relapse; assessment should consider chronic pain and biomechanical contributors alongside objective evidence of disease activity.

2. Psychosocial complexity may predict transition risk more strongly than disease severity and should be actively explored through structured adolescent assessment.

3. Transition should be viewed as a longitudinal process rather than a single transfer event. Early planning, MDT working, specialist nurse continuity, youth support workers and enhanced pathways may improve continuity and engagement.

4. Adolescents with safeguarding concerns, mental health history or unstable family circumstances may require tailored transition support and repeated opportunities for engagement.

5. Transition clinics provide an opportunity to address wider adolescent health needs including reproductive health, contraception counselling and medication safety alongside disease management.

6. Well-coordinated regional transition pathways are important in preparing vulnerable young people for successful engagement with adult rheumatology services and reducing the risk of disengagement during transfer.

